# The orisome: structure and function

**DOI:** 10.3389/fmicb.2015.00545

**Published:** 2015-06-02

**Authors:** Alan C. Leonard, Julia E. Grimwade

**Affiliations:** Department of Biological Sciences, Florida Institute of Technology, MelbourneFL, USA

**Keywords:** *oriC*, DnaA, DNA replication, replication origin, DNA binding proteins, orisomes, pre-replication complexes

## Abstract

During the cell division cycle of all bacteria, DNA-protein complexes termed orisomes trigger the onset of chromosome duplication. Orisome assembly is both staged and stringently regulated to ensure that DNA synthesis begins at a precise time and only once at each origin per cycle. Orisomes comprise multiple copies of the initiator protein DnaA, which oligomerizes after interacting with specifically positioned recognition sites in the unique chromosomal replication origin, *oriC*. Since DnaA is highly conserved, it is logical to expect that all bacterial orisomes will share fundamental attributes. Indeed, although mechanistic details remain to be determined, all bacterial orisomes are capable of unwinding *oriC* DNA and assisting with loading of DNA helicase onto the single-strands. However, comparative analysis of *oriC*s reveals that the arrangement and number of DnaA recognition sites is surprisingly variable among bacterial types, suggesting there are many paths to produce functional orisome complexes. Fundamental questions exist about why these different paths exist and which features of orisomes must be shared among diverse bacterial types. In this review we present the current understanding of orisome assembly and function in *Escherichia coli* and compare the replication origins among the related members of the Gammaproteobacteria. From this information we propose that the diversity in orisome assembly reflects both the requirement to regulate the conformation of origin DNA as well as to provide an appropriate cell cycle timing mechanism that reflects the lifestyle of the bacteria. We suggest that identification of shared steps in orisome assembly may reveal particularly good targets for new antibiotics.

## Introduction

As the commitment step for proliferation, initiating new rounds of chromosomal DNA synthesis is arguably the paramount event in the life of a bacterial cell. It is also a precarious step, which must rely on sophisticated regulatory mechanisms to ensure that new replication forks are established with sufficient time and number to provide every daughter cell with at least one complete genome copy, regardless of cellular growth rate. Many of these regulatory mechanisms are focused on orisomes, the large multimeric complexes of the bacterial initiator protein, DnaA, that assemble along the unique chromosomal replication origin, *oriC*.

DnaA is a highly conserved protein whose activity is regulated by the binding and hydrolysis of ATP, with initiation requiring the ATP-bound form (Sekimizu et al., 1987). The crystal structure of a truncated version (domains III and IV) of *Aquifex aeolicus* has been determined (Erzberger et al., 2002, 2006), revealing that DnaA is not only conserved among bacteria, but also has structural similarity to archaeal and eukaryotic initiatior proteins. Additionally, several laboratories have used reverse genetics to introduce targeted mutations in Domains I, III, and IV, and use of these mutants has revealed key roles for these domains in DnaA recruitment (I), binding (IV), oligomerization (I, III), and helicase loading (I, III; see below and representative reviews; Kaguni, 1997; Zawilak-Pawlik et al., 2005; Mott and Berger, 2007; Ozaki and Katayama, 2009; Leonard and Grimwade, 2011).

Insightful studies on how orisomes assemble, function, and are regulated have come from many laboratories over the course of several decades, mostly using *Escherichia coli*. Cloning of *E. coli oriC* onto plasmids (minichromosomes) allowed determination that all instructions for normal orisome assembly are contained in the *oriC* nucleotide sequence (Leonard and Helmstetter, 1986). Sequencing the cloned *oriC* (Meijer et al., 1979) identified repeated 9 mer sequences that were determined to be DnaA recognition site (Fuller et al., 1984; Matsui et al., 1985), providing the framework that allowed analysis of how DnaA contacted DNA (Speck et al., 1997; Fujikawa et al., 2003; Yoshida et al., 2003). Seminal studies done by the Kornberg lab provided evidence that initiation could be studied *in vitro* using crude extracts and purified proteins (Fuller et al., 1981; Kaguni and Kornberg, 1984) and revealed the major stages of orisome assembly (Sekimizu et al., 1988). More recently, high-resolution mapping of DnaA-*oriC* interactions (McGarry et al., 2004; Rozgaja et al., 2011), biochemical analysis of ordered orisome assembly (Margulies and Kaguni, 1996), and characterization of key subassemblies (Ozaki and Katayama, 2012; Ozaki et al., 2012) have given a clearer picture of how orisomes assemble in *E. coli*, and, to a lesser extent, in other bacterial types [reviewed in Wolanski et al. (2014)]. However, fundamental questions remain about the relationship between DnaA oligomer formation and the DnaA-directed changes in DNA conformation necessary to unwind the origin, as well as the manner by which orisome assembly is precisely timed in during cell cycle.

The majority of studies on orisomes have been done *in vitro*, using either DNA fragments or plasmid templates. Plasmids are also useful tools for site-specific mutagenesis, which is essential for dissection of the roles of individual DnaA recognition sites and other *oriC* sequence elements. However, when the effects of *oriC* mutations on orisome function were examined *in vivo*, it became apparent that mutations in cloned *oriC* do not always have the same effect when placed into the chromosomal context (Weigel et al., 2001). Although the regulatory factors and timing during the cell cycle are identical for plasmid and chromosomal *oriC*s, there are obvious differences in DNA topology, and intracellular location (Niki and Hiraga, 1999). Most importantly, functional studies of mutant cloned *oriCs* were usually performed in hosts that harbored wild-type *oriC* on their chromosomes, setting up a potential competition for available DnaA (Grimwade et al., 2007). Under these conditions the winner ultimately excludes the *oriC* that loses (does not meet the threshold requirement for DnaA) and causes either death of the host or replacement of the wild-type chromosomal origin with the mutant cloned version. Thus it seems preferable to dissect orisome function *in vivo* by introducing mutations in chromosomal *oriC*, but this has, in the past, been a difficult, and labor-intensive process.

**FIGURE 1:**
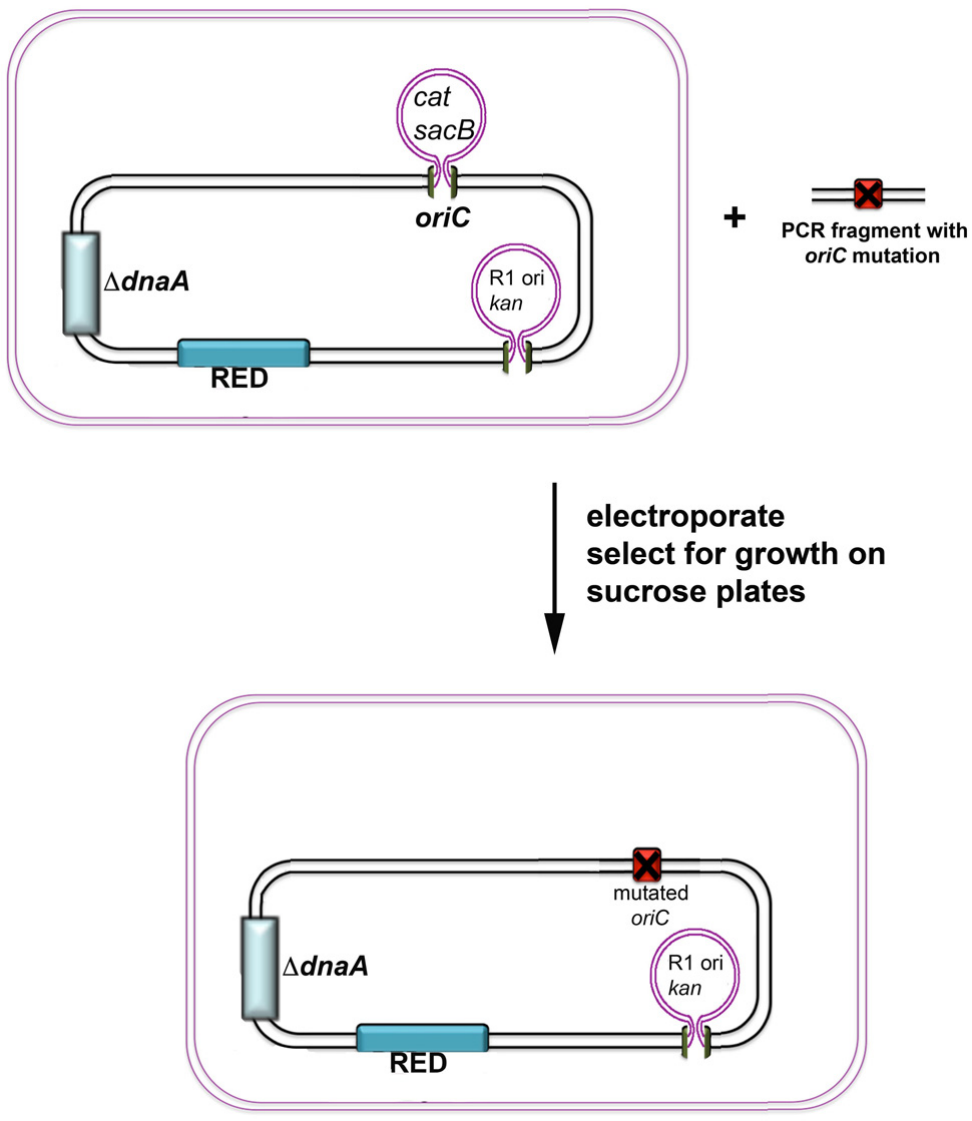
**Scheme of the *oriC*-specific recombineering method.** Recombineering strains harbor *sacB* and *cat* genes replacing all of the *oriC* sequence, inducible genes encoding the lambda RED system, and a plasmid origin of replication (R1*ori*) linked to a kanamycin resistance determinant. The strain also has a deletion in the *dnaA* gene. For insertion of *oriC* mutants into the chromosome, a PCR fragment carrying the desired mutation is electroporated to transform cells in which the RED system was induced. Recombination results in replacement of the *sacB* and *cat* genes with the mutated *oriC*. Successful recombineering confers the ability to grow in the presence of sucrose, and sensitivity to chloramphenicol.

The development of *E. coli* recombineering strains that express inducible lambda phage recombination proteins (RED strains; Datsenko and Wanner, 2000; Sharan et al., 2009) has streamlined the introduction of mutations into chromosomal *oriC* or anywhere else on the genome. Short PCR fragments containing mutant *oriC* can now be easily inserted as perfect replacements on the chromosome without any additional genetic alterations. Furthermore, these replacements can be performed in strains whose chromosomal replication is under the control of an DnaA-independent, integrated R1 plasmid (Hansen and Yarmolinsky, 1986; Koppes and Nordström, 1986) so that even mutations that render *oriC* non-functional can be introduced into a DnaA null strain (Kaur et al., 2014). Recently, recombineering strains were constructed with the *sacB* gene from *Bacillus* within *oriC* to permit selection for *oriC* replacements on sucrose-containing plates (only strains that lack *sacB* will grow; **Figure 1**). Since the *oriC* replacement strains still contain an integrated R1 plasmid origin, it is possible to quickly test for function of the mutant *oriC* by measuring cell viability in the presence of a plasmid that expresses both DnaA and the R1*ori* repressor (*copA*) RNA (Stougaard et al., 1981). Once the mutation is confirmed by nucleotide sequence analysis, functional *oriC*s can be transduced into strains with clean genetic backgrounds to study the effect of the mutation on cell growth or cell cycle timing.

*Escherichia coli* recombineering strains also show promise for the development of novel heterologous systems that will allow *in vivo* examination of DnaA-*oriC* interactions that are difficult to perform in native strains, particularly for slow growers or pathogens. Since *E. coli* DnaA is not required to drive chromosome replication in the recombineering strains, any heterologous *oriC* and DnaA combination (and any other proteins associated with orisome function) can be introduced at any chromosomal location desired.

## Getting Started: The Multifunctional Bacterial Origin Recognition Complex (bORC)

**FIGURE 2:**
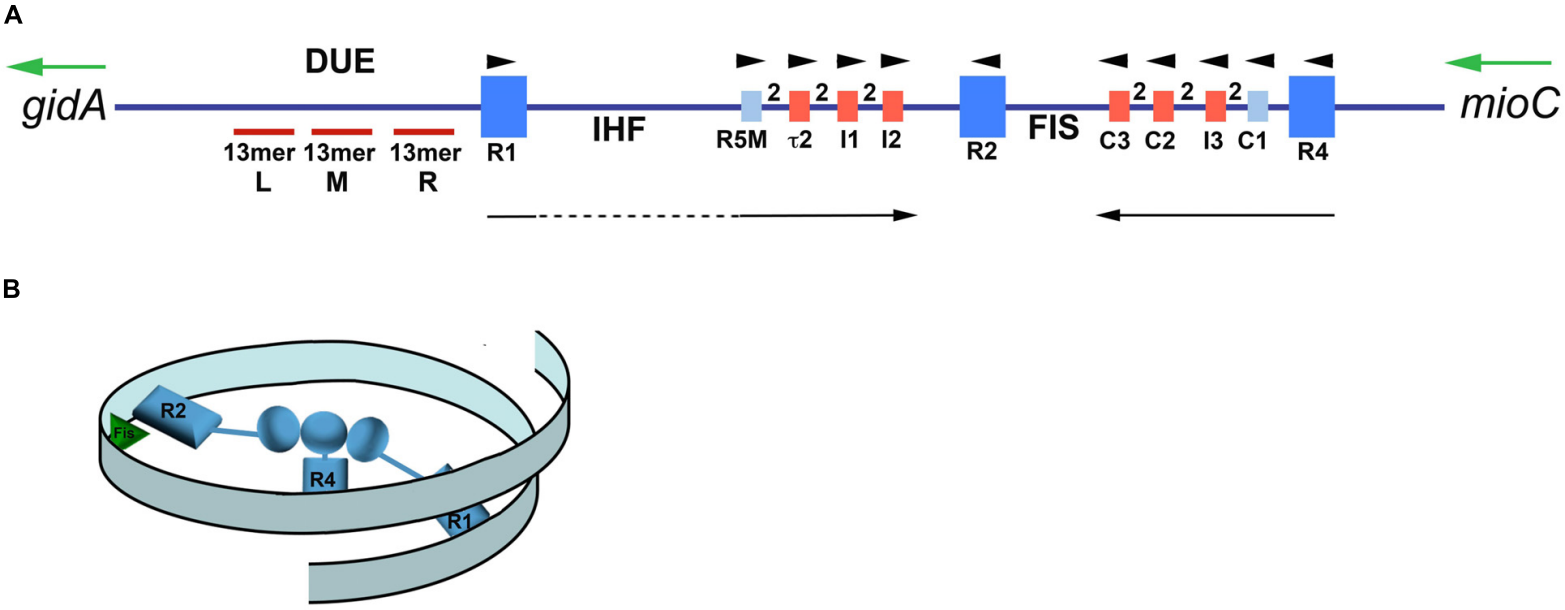
**Map of *Escherichia coli oriC* and conformation of bORC. (A)** The *oriC* region is mapped, showing positions of binding sites for DnaA, IHF, and Fis, as well as the right (R), middle (M), and left (L) 13mer sequences in the DNA unwinding element (DUE). The three high-affinity sites R1, R2, and R4 are designated by royal blue squares, and the low-affinity sites are marked by small light blue or red rectangles. The red rectangles designate sites that preferentially bind DnaA–ATP, while the sites marked by light blue rectangles bind both nucleotide forms of DnaA equivalently. Small arrowheads show orientation of sites, the two between sites indicates the number of bp separating the sites. Arrows under the map indicate growth direction of DnaA oligomers. The dotted line marks that the oligomer does not span the region between R1 and R5M. Two genes, *gidA* and *mioC,* flanking *oriC* are shown, with the green arrows marking the direction of transcription. **(B)** Proposed looped conformation of bORC in *E. coli*. *OriC* DNA (ribbon) is constrained by DnaA (gray-blue figures in center of loop) bound at R1, R2, and R4 sites, as labeled. Interaction among the three bound DnaAs proposed to be via domain I. The green triangle represents Fis bound to its cognate site.

In order to build orisomes reproducibly during every cell division cycle, there needs to be an invariant starting scaffold that is capable of not only recruiting additional orisome components for later stages of assembly, but also of arranging origin DNA into an appropriate configuration that prohibits DnaA-independent *oriC* DNA unwinding. This latter feature may be unexpected, but when they are in the supercoiled topology that is required for origin function (Funnell et al., 1986; Von Freiesleben and Rasmussen, 1992), *oriC* templates contain single-stranded DNA in the absence of any associated protein (Kowalski and Eddy, 1989). Unwinding is observed in an A-T rich region (DNA Unwinding Element, or DUE; **Figure 2A**; Kowalski and Eddy, 1989), which is identical to the region which unwinds following orisome assembly (Bramhill and Kornberg, 1988). In *E. coli*, the DUE contains three 13 mer repeats with consensus sequence 5′-GATCTnTTnTTTT-3′, although the nucleotide sequences of DUEs can be quite variable among other bacterial types (Zawilak-Pawlik et al., 2005).

To avoid premature unwinding, bacterial *oriC* is usually not naked in cells, but rather is bound to DnaA at high affinity (K_d_ in the range of 4–20 nM) recognition sites (5′-TTATC/ACACA-3′), via a helix-turn-helix (HTH)-type DNA binding motif located within the C-terminal domain (domain IV; Speck et al., 1997; Erzberger et al., 2002; Fujikawa et al., 2003). Three such sites (R1, R2, and R4) are found in *E. coli oriC* (**Figure 2A**). DnaA occupies these sites as soon as they becomes accessible, usually after the origin DNA is replicated, and remains bound to them throughout the majority of the cell cycle (Samitt et al., 1989; Nievera et al., 2006). One consequence of this binding is prevention of strand separation, since unwinding of the *E. coli* DUE, detected by susceptibility to single-strand DNA-specific endonucleases, is completely eliminated when DnaA occupies all three high affinity sites. Endonuclease cutting returns when any single site is unoccupied as a result of mutation (Kaur et al., 2014). This observation is consistent with the idea that supercoiled *oriC* is constrained by the direct interaction among the bound DnaA molecules. Although the nature of the interaction region is not yet known, a logical candidate is a globular K-homology (KH)-type fold mapped to the N-terminus (domain I; Weigel et al., 1999; Simmons et al., 2003). Domain I is attached to the rest of DnaA by a long flexible linker (domain II; Nozaki and Ogawa, 2008) that could facilitate association of bound DnaA molecules. The high affinity recognition sites in *E. coli* are widely separated (**Figure 2A**), and even using the flexible linker, it is difficult to conceive of a way for all the bound DnaA molecules to interact without the formation of DNA loops. An example of a looped bORC is shown in **Figure 2B**, although this structure is still hypothetical. Since the orisome of *E. coli* appears as a nucleosome-like structure when observed using the electron microscope (Crooke et al., 1993), an intriguing possibility is that as orisome assembly progresses, newly recruited DnaA is added to loosely looped DNA, and then forms oligomers that tighten the loops to form a quasi-nucleosome.

The minimal trimeric DnaA complex bound to *E. coli oriC* is temporally equivalent to the persistent and well-characterized hexameric eukaryotic origin recognition complex (ORC) that resides at eukaryotic replication origins (Duncker et al., 2009), and we will refer to the bacterial version as bORC. An important difference is that in eukaryotes, ORC is preassembled prior to interacting with replication origins and specific nucleotide motifs are not usually recognized (Bell, 2002; Kawakami and Katayama, 2010). Although DnaA can aggregate in solution, *oriC* recognition sites are occupied by monomers (Schaper and Messer, 1995; Weigel et al., 1997), with DnaA–DnaA interactions taking place after monomers have bound to DNA.

In additional to being temporally analogous, bORC has functional similarity to the eukaryotic ORC. Like ORC, which recruits additional components of the eukaryotic pre-replication complex, the DnaA in bORC also acts as a scaffold for the recruitment of more copies of DnaA to form subsequent stages of the orisome (Miller et al., 2009; Smits et al., 2011). DnaA molecules are recruited to bORC via domain I (Miller et al., 2009) and a separate DnaA oligomerization region in domain III, encompassing the AAA+ (ATPases associated with various cellular activities) fold (Felczak and Kaguni, 2004; Kawakami et al., 2005). Mutations in either domain I or III that abolish DnaA–DnaA interactions also prevent progression of orisome assembly beyond bORC.

Although all three high affinity sites in *E. coli oriC* are required to constrain the origin, any combination of two high affinity sites are sufficient to assemble orisomes that are functional, albeit with timing defects (Kaur et al., 2014). However, orisomes lacking either R1 or R4 become dependent on two DNA bending proteins that bind to *oriC* (Fis and IHF, **Figure 2** and see below; Kaur et al., 2014), suggesting that there are conformational requirements for bORC. The ability of orisomes to function without DnaA-binding to R1 also counters previous models that proposed that R1 is essential for DnaA-dependent DNA helicase loading (Speck and Messer, 2001; Soultanas, 2012), and this finding suggests that either R1 is not required for this step, or that another component of the orisome can provide a back-up function when R1 is inactivated.

The nature of bORC in other bacterial types is not well characterized. Clustering of high affinity DnaA recognition sites near A-T rich regions is used as a criteria for mapping bacterial replication origins on newly sequenced genomes (Gao and Zhang, 2007), but the numbers, positions, and orientations of these sites is remarkably variable. Most bacteria carry between 3 and 8 high affinity DnaA recognition sites, but *oriC* geography is distinctive for each genus (Marczynski and Shapiro, 1993; Zawilak-Pawlik et al., 2005; Gao and Zhang, 2007; Leonard and Mechali, 2013; see also **Figure 4**). While larger origins usually have higher numbers of high affinity recognition sites, there are, as yet, no hard and fast rules that can be used to predict the configuration of initiator binding sites in *oriC*s. For example, the thermophile *Thermus thermophilus* carries a 300 bp *oriC* with 13 consensus or near consensus recognition sites (Schaper et al., 2000). In *Caulobacter*, there is only one high affinity DnaA recognition site, but many recognition sites for the regulatory protein CtrA (Marczynski and Shapiro, 1992). This arrangement allows the initiation step to remain DnaA-dependent, but under the control of an additional regulator that ensures the initiation step is restricted to a particular stage of the cell cycle. In some bacteria, for example, *Helicobacter* (Donczew et al., 2012), *Mycoplasma* (Cordova et al., 2002; Lartigue et al., 2003), and *Bacillus* (Moriya et al., 1999), there are two separated clusters of high affinity DnaA recognition sites. This arrangement produces a bipartite configuration whereby DnaA bound at each cluster can interact, but this interaction becomes dependent on DNA bending.

## Filling the Gaps: Ordered Orisome Assembly is Determined by Low Affinity DnaA-*oriC* Interactions

Since DnaA occupies high affinity sites throughout the cell cycle, mechanisms regulating progression of orisome assembly beyond bORC must be focused on lower affinity DnaA-*oriC* interactions. Low affinity DnaA recognition sites are mapped in a variety of bacterial *oriC*s (Charbon and Lobner-Olesen, 2011; Leonard and Mechali, 2013) but these sites are often difficult to identify because they deviate substantially (two or more bases) from the consensus sequence. Thus, direct measurement of DnaA contacts with these sites is often required to confirm their role in orisome assembly (Rozgaja et al., 2011). In *E. coli*, the recognition sites that are not bound in the bORC have at least a 50-fold lower DnaA binding affinity than do R1, R2, and R4 (Schaper and Messer, 1995). Most importantly, low affinity DnaA recognition sites cannot become occupied unless DnaA is recruited by bound DnaA at a nearby site, and this assistance requires DnaA’s domain I (Schaper and Messer, 1995; Rozgaja et al., 2011). For this reason, it is not surprising that the low affinity DnaA recognition sites in *E. coli oriC* lie in the two DNA gaps (Leonard and Grimwade, 2010) flanked by high affinity recognition sites (see **Figure 2A**). The arrangement of the low affinity recognition sites within these gaps was unexpected, but provides insight into how cooperative binding leading to DnaA occupation of low affinity sites is achieved. Each of two distinct arrays contains four sites, one in each half of *oriC* (**Figure 2A**; R5M, t2, I1, and I2 in the left half and C3, C2, I3, and C1 in the right half; Rozgaja et al., 2011). Notably, each low affinity site within an array is separated from its neighbor by exactly two base pairs, which positions DnaA on the same face of the DNA helix. All the recognition sites in an individual array face in the same direction in *E. coli*, and the arrays in each half of *oriC* are also oriented in opposite directions relative to one another (Rozgaja et al., 2011; see **Figure 2**). This arrangement allows a DnaA bound to a high affinity site to assist loading at the proximal low affinity site, with the other sites in the array being filled by progressive cooperative binding between two low affinity sites (Rozgaja et al., 2011).

Spacing between adjacent DnaA recognition sites is critical for these cooperative interactions (Hansen et al., 2007). Since the spacing between high and low affinity sites in *oriC* varies among bacterial types, and since domain I interactions play a key role in cooperative binding at *oriC* (Miller et al., 2009), this stringency in spacing implies that the length of the flexible linker region in DnaA’s domain II may contribute to the efficiency of orisome assembly. Consistent with this idea, deletions that shorten domain II result in an under-initiation phenotype (Molt et al., 2009). Thus, it is not surprising that the length and amino acid sequence of domain II is the least conserved property of DnaA, and it is likely that among different bacterial types, there is a direct relationship between linker length/flexibility and placement of recognition sites in *oriC* (Nozaki and Ogawa, 2008).

It should be noted that in some bacterial types, the affinity for DnaA may not be as high as is measured for R1 and R4 in *E. coli oriC*, and many closely spaced consensus recognition sites may be required for co-operative DnaA binding, as exemplified by the *oriC* of *Thermus* (Schaper et al., 2000), and *Streptomyces* (Jakimowicz et al., 2000). Specific spacing requirements will become more apparent as new low affinity sites are identified in different bacterial origins, and this will necessitate substantial revisions of existing *oriC* maps, as has happened for *E. coli* (Leonard and Grimwade, 2011). It is also important to not rule out the role of DNA topology in the ability of DnaA to access its recognition sites, and supercoiling dependent DnaA binding in *oriC* is reported for the *oriC* of *Helicobacter pylori* (Donczew et al., 2014).

## Dynamic Conformational Switching in *oriC*: Regulation of Staged Orisome Assembly

**FIGURE 3:**
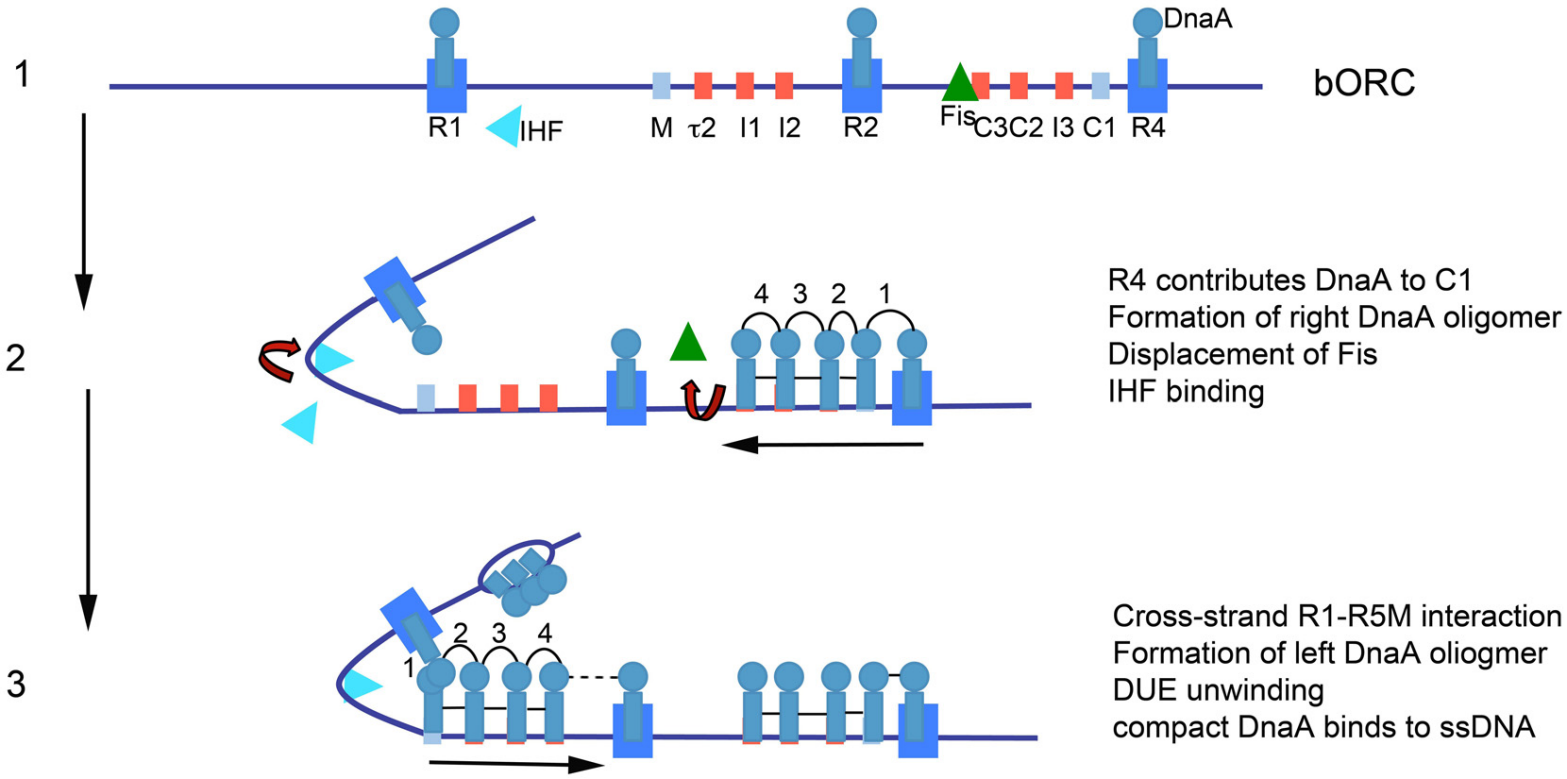
**Model of staged orisome assembly.** Stage 1 (bORC): after initiation of chromosome replication, DnaA rebinds to high affinity R1, R2, and R4 sites. Fis is also bound at this stage, but IHF is not. Low affinity sites are unoccupied. Stage 2: DnaA bound to R4 recruits DnaA for binding to C1. DnaA then progressively fills the remaining arrayed sites, forming an oligomer in the gap region between R2 and R4. The DnaA oligomer displaces Fis, and loss of Fis allows IHF to bind to its cognate site. Stage 3: the bend induced by IHF binding allows DnaA, recruited by R1, to bind to R5M, and form a cross-strand DnaA interaction. A DnaA oligomer then progressively grows toward R2, bound to arrayed low affinity sites, and anchored by R2. In this configuration, oriC DNA is unwound in the DUE, and DnaA in the form of a compact filament binds to the ssDNA.

The precise positioning and spacing of DnaA recognition sites encoded into *E. coli oriC,* described above, provides the instructions for ordered orisome assembly. Starting from bORC, the first cooperative binding that should take place, based on proximity, is between DnaA bound at R4 and the adjacent low affinity site C1 (**Figure 2A**), which nucleates a DnaA oligomer that grows by progressive binding of the low affinity sites in the gap between R4 and R2 (Rozgaja et al., 2011; **Figure 3**). Given the positions and orientations of DnaA recognition sites in the left half of *oriC*, assembly of a similar DnaA oligomer in the gap region between R1 and R2 was predicted and then detected (Rozgaja et al., 2011). Experimental evidence is consistent with both left and right oligomers growing toward R2 (Rozgaja et al., 2011), and this arrangement means that the DnaA occupying R2 primarily acts to anchor the converging oligomers. Complete loss of DnaA binding at R2 is well tolerated (Weigel et al., 2001) so anchoring of the DnaA oligomers does not appear to be a critical step in orisome assembly. The observation that *E. coli oriC* retains function in the absence of binding to R1 or R4 implies that the DnaA occupying R2 is capable of nucleating oligomers, and this was shown to be true (Kaur et al., 2014), although R2-bound DnaA does not nucleate as efficiently as the DnaA occupying the peripheral sites, particularly in the right half of *oriC.*

Despite the appealing symmetry of converging DnaA oligomers, there is an obvious difference in the nucleation of oligomers from DnaA bound at R1 and R4, due to their distance from their proximal sites. Extension of DnaA from R4 is easily accomplished, due to close spacing (3 bp) of R4 and C1 (Rozgaja et al., 2011), but there is a 45 bp gap between R1 and the nearest low affinity recognition site, R5M, and there is evidence that DnaA oligomers nucleated at R1 do not extend into this region (Rozgaja et al., 2011). Rather, to span this 45 bp space, *E. coli oriC* DNA is bent to bring R1 and R5 into proximity, and the DnaA oligomer that grows by progressive binding of DnaA monomers to the left array of low affinity sites is most likely nucleated by cross-strand DnaA–DnaA interactions via domain I (**Figure 3**). A DNA bending protein, integration host factor (IHF) recognizes a site placed in the gap between R1and R5M (Polaczek, 1990; **Figure 2A**) and facilitates the interaction between DnaAs bound at R1 and R5M. IHF binding is not essential for R1 to nucleate a DnaA oligomer or for assembly of functional orisomes (Von Freiesleben et al., 2000; Weigel et al., 2001), presumably because the DNA between R1 and R5M is intrinsically flexible, but loss of IHF binding results in perturbed initiation timing.

In rapidly growing cells, assembly of oligomers nucleated by DnaA bound to R1 and R4 is nearly coincident, and correlates closely with the time that IHF binding to its cognate site in *oriC* can be detected, and with the time of initiation of DNA synthesis (Cassler et al., 1995). Thus, *in vivo*, assembly of the right and left DnaA oligomers must be both coordinated and precisely timed. This coupled oligomer assembly appears to be critical, since any loss of precisely ordered orisome assembly causes defects in initiation efficiency or timing (Kaur et al., 2014), especially for bacteria undergoing rapid growth, where new rounds of DNA replication must initiate before previous rounds are completed. In rapidly growing *E. coli* where multiple copies of *oriC* exist, all origins are activated synchronously to ensure that replication is completed for all chromosomes at the correct time (Skarstad et al., 1986).

To ensure orderly DnaA oligomerization, *E. coli* uses a DnaA-dependent switch mechanism that is built around the successive activity of IHF and another DNA bending protein, Fis (Factor for inversion stimulation), whose recognition site is placed in the gap regions between R2 and C3 (**Figure 2A**; Filutowicz et al., 1992; Ryan et al., 2004). In rapidly growing *E. coli*, Fis binds to bORC and places a bend in the constrained bORC loop (Cassler et al., 1995; **Figure 3**). This bend acts as a long distance inhibitor of IHF binding (Ryan et al., 2004), most likely because it prevents additional bending within the loop (Kaur et al., 2014). Close to the time of initiation, DnaA bound to R4 nucleates assembly of the DnaA oligomer bound to the right array of low affinity sites. Fis is displaced from its site during this process (Ryan et al., 2004; **Figure 3**), and it is possible that interactions between high affinity sites are also broken, although this has yet to be determined. Displacement of Fis allows IHF to bind and bring DnaA bound to R1 into proximity to R5M (Cassler et al., 1995), resulting in nucleation of the left side oligomer (Ryan et al., 2004; **Figure 3**). Full occupation of *oriC* with DnaA is coincident with origin unwinding in the DUE (Grimwade et al., 2000).

The possibility that DNA bending itself may promote unwinding of the DUE was suggested by the timing of the IHF-induced bend. However, in the absence of DnaA, IHF is not capable of creating a single-strand DNA bubble (Grimwade et al., 2000), so some feature of the left oligomer is also required. One possibility is that DNA bending is sufficient for unwinding, but the unwinding is not sustainable, and so the left oligomer is needed to stabilize the unwound single-stranded DNA, and/or participate in DNA helicase loading (Duderstadt et al., 2011; Ozaki and Katayama, 2012). It should be noted that the IHF-induced bend places the DUE in closer proximity to the DnaA bound to the left array of low affinity sites (**Figure 3**). Alternatively, if bending alone is not sufficient to separate DNA strands in the DUE, then the left oligomer may play a direct role in unwinding, and this has been suggested based on *in vitro* studies (Ozaki et al., 2012). How left oligomer formation might mediate strand separation is not clearly understood. One model suggests that the DnaA oligomer induces positive supertwists in *oriC* DNA that result in compensatory negative supercoiling that promotes DNA unwinding of the DUE (Erzberger et al., 2006; Zorman et al., 2012). There is less evidence for a role of the right oligomer in unwinding, since *oriC* deletion mutants lacking the right half of *oriC* are viable at slow growth rates, albeit with severely perturbed initiation (Stepankiw et al., 2009). However, the right oligomer cannot be ruled out as a contributor to events in the late stages of orisome assembly, particularly in the loading of DnaB helicase (Ozaki et al., 2012).

The nucleation of DnaA oligomers from high affinity sites is a critical step, and requires domain I-domain I interactions between DnaA molecules (Miller et al., 2009). Given the importance of this stage of orisome assembly, it is logical that it would be a target for regulation. While this remains to be determined, there are several good candidates for regulatory factors. In particular, DiaA (Ishida et al., 2004) and its structural analog HobA (Natrajan et al., 2009; Zawilak-Pawlik et al., 2011), are both positive effectors of orisome assembly, and stimulate the assembly of DnaA oligomers. Both proteins form homotetramers that bind to domain I directly (Keyamura et al., 2009; Zawilak-Pawlik et al., 2011). *E. coli* DiaA interacts with a subgroup of DnaA molecules binding to *oriC,* although the position of these molecules is not known (Keyamura et al., 2007). DnaB helicase and DiaA interact with DnaA domain I at the same location, suggesting that DiaA plays a negative role in the regulation of DNA helicase loading (Keyamura et al., 2009). Although the protein is dispensable, *E. coli* mutants of DiaA show delayed initiation of chromosome replication (Ishida et al., 2004). HobA is an essential factor in *H. pylori* (Zawilak-Pawlik et al., 2007), and tetramers of HobA are required for domain I-domain I DnaA oliogmerization (Natrajan et al., 2009). Despite their structural similarity, DiaA and HobA are not interchangeable suggesting a high degree of specificity for the DnaA regulating factors among bacterial types (Zawilak-Pawlik et al., 2011), possibly due to differences in domain I.

Some factors that interact with DnaA domain I are repressors of chromosome replication. This is the case for the *E. coli* ribosomal protein L2 (Chodavarapu et al., 2011), the *E. coli* starvation protein, Dps (Chodavarapu et al., 2008), and the sporulation-related regulator of *Bacillus subtilis*, SirA (Rahn-Lee et al., 2011). SirA is an important regulator of ploidy in *Bacillus* and during sporulation, (Wagner et al., 2009) cells lacking this protein will over-initiate the replication of their chromosomes.

## Dissecting the Role of DnaA–ATP in Orisome Assembly and in Timing of Initiation of Chromosome Replication

New rounds of chromosome replication in most bacteria are dependent on newly synthesized DnaA–ATP and DnaA–ATP is commonly considered to be the “active form” of the initiator. *In vitro* studies also identified an additional requirement for ATP in the mM range (Sekimizu et al., 1987); this is much higher than is needed for DnaA to bind ATP (μM range; Sekimizu et al., 1987), and the reason for this requirement has not yet been determined. Additionally, experimental evidence from both *in vitro* and *in vivo* studies indicates that some DnaA–ADP is permitted in a functional orisome in *E. coli* (Yung et al., 1990; McGarry et al., 2004; Grimwade et al., 2007), and it is not yet fully understood what features of orisome assembly and function specifically require DnaA–ATP. One possibility is that DnaA–ATP forms a specific structure on DNA that is required for orisome function. In the absence of DNA, a truncated version (domains III and IV) of *Aquifex aeolicus* DnaA–ATP assembles into an open ended, compact, right handed, helical filament (Erzberger et al., 2006). In this configuration, an arginine finger in one molecule’s domain III contacts the ATP bound to the adjacent protomer. The DNA-binding domain (domain IV) also folds up to contact domain III in the helical filament, implying that a crosstalk mechanism exists between domains III and IV (Duderstadt et al., 2010). These compact DnaA–ATP filaments can interact directly with single-stranded DNA through a central channel formed by domains III and IV. Two distinct roles, in helicase loading and unwinding, have been proposed for the compact DnaA–ATP filament. Evidence to support these roles comes from *in vitro* studies, where this structure has been reported to bind the helicase loader, DnaC, and possibly mediate asymmetric loading of the replicative helicase, DnaB (Mott et al., 2008). The filament has also been shown to melt short DNA duplexes, presumably by stretching the helix (Duderstadt et al., 2011). However, the compact filament has very low affinity for double-stranded DNA (Duderstadt et al., 2010), and it is unclear how its assembly could be started in the DUE unless the first protomer was recruited to DNA that was already unwound.

Although the crystal structure of DnaA–ATP oligomers is compatible with only single-stranded DNA binding, there is also experimental evidence that DnaA–ATP filaments assemble on double-stranded DNA in the gap regions between high affinity site, where they are proposed to mediate unwinding (left gap region) and assist in helicase loading (right gap region; Ozaki and Katayama, 2012). DnaA–ATP oligomers have also been shown to play a key role in *Bacillus* orisome function *in vivo*, although it is not known where in *oriC* these oligomers are formed. Oligomerization of *Bacillus* DnaA–ATP is stimulated by either single-stranded DNA or double-stranded DNA (Scholefield et al., 2012), but stimulation does not require site-specific DnaA binding and the role of DNA in this reaction remains unclear.

It is also not clear if the oligomers formed on double-stranded DNA must contain only DnaA–ATP. In *E. coli*, DnaA–ADP and a mutant version of DnaA (R285A), defective in domain III oligomerization, do not fill low affinity binding sites (McGarry et al., 2004; Kawakami et al., 2005), but it is not yet known if this is because only DnaA–ATP can participate in the DnaA–DnaA interactions required for cooperative binding, or because the nucleotide sequence of some of the low affinity 9 mer recognition sites preferentially binds DnaA–ATP (McGarry et al., 2004; Saxena et al., 2011). In support of the latter idea, a single base pair in the 9 mer recognition sequences of I2 and I3 is sufficient to convert these sites into ones that bind both nucleotide forms, without increasing their affinity for DnaA–ATP (McGarry et al., 2004). It is not known if low affinity sites that discriminate between DnaA’s nucleotide forms are common among different bacterial types.

The structure of the DnaA oligomer associated with double-stranded DNA has not been determined. Duderstadt et al. (2010) suggested that DnaA–ATP forms a less compact oligomer on double-stranded DNA, and single molecule studies examining interaction of DnaA with *E. coli oriC* are consistent with DnaA–ATP, but not DnaA–ADP, forming a right handed helical filament on double-stranded DNA (Zorman et al., 2012). However, since *E. coli oriC* was used as the DNA substrate for the single molecule studies, it is possible the DnaA–ADP did not oligomerize because it was not bound to the origin fragment.

Despite the lack of clarity on how DnaA–ATP participates in orisome function and structure, it is clear that properly timed initiations require tight control of DnaA–ATP binding to *oriC*. In *E. coli*, multiple mechanisms, reviewed in Katayama et al. (2010) ensure that the availability of active initiator at *oriC* fluctuates appropriately during the cell cycle (Kurokawa et al., 1999), and that full occupation of *oriC* by DnaA is restricted to a short period of time during the cell cycle. These mechanisms, described below, include: (1) converting active DnaA–ATP into the inactive ADP-bound form, (2) titrating DnaA by binding it to sites outside *oriC*, (3) blocking DnaA access to *oriC*, and when necessary, (4) reactivating DnaA–ADP into active DnaA–ATP.

Mechanisms that inactivate DnaA–ATP activity are often coupled to the elongation phase of DNA replication. In *E. coli*, conversion of DnaA–ATP to DnaA–ADP is accomplished primarily by interactions with a replisome-associated protein, Hda, which stimulates DnaA’s hydrolytic activity in a process termed regulatory inactivation of DnaA (RIDA; Katayama et al., 1998; Katayama and Sekimizu, 1999; Keyamura and Katayama, 2011). Since the nucleotide exchange rate for DnaA in many Gram positives is much higher than in Gram negatives, stimulating DnaA’s hydrolysis activity is apparently not an effective regulatory mechanism in all bacteria (Bonilla and Grossman, 2012), and consistent with this idea, Hda is not a conserved protein.

In several bacterial types, DnaA is titrated away from *oriC* by individual high affinity recognition sites dispersed around the chromosome (Roth and Messer, 1998; Christensen et al., 1999), or by localized clusters of DnaA recognition sites at one, or more positions on the genome. One well-characterized cluster in *E. coli*, *datA*, resides over 450,000 bp away from *oriC* (Kitagawa et al., 1996; Ogawa et al., 2002) and can titrate large amounts of DnaA–ATP. At *datA*, DnaA assembles into a higher order complex that promotes DnaA–ATP hydrolysis (Kasho and Katayama, 2013). Clusters of DnaA are also found at eight intergenic regions on the *Bacillus* chromosome (Ishikawa et al., 2007). The conserved YabA protein found in several Gram-positive bacteria may represent a replisome-associated DnaA titration mechanism (Noirot-Gros et al., 2006; Soufo et al., 2008), but YabA is also reported to inhibit cooperative DnaA binding to *oriC* (Merrikh and Grossman, 2011; Scholefield and Murray, 2013).

Multiple factors in many different bacterial types are reported to bind to *oriC* and either block DnaA accessibility or affect its cooperative binding/oligomerization. These include CtrA in *Caulobacter* (Quon et al., 1998), AdpA in *Streptomyces* (Wolanski et al., 2012), MtrA in *Mycobacteria* (Rajagopalan et al., 2010), HP1021 in *Helicobacter* (Donczew et al., 2015), as well as SirA (Rahn-Lee et al., 2011), Soj (Scholefield et al., 2012), and DnaD (Bonilla and Grossman, 2012; Scholefield and Murray, 2013) in *Bacillus*. The master regulator of *Bacillus* sporulation, Spo0A, is also reported to bind to *oriC,* and block rounds of chromosome replication (Boonstra et al., 2013). In *E. coli*, direct blocking of access to low affinity sites is performed during every cell cycle by the sequestration protein, SeqA (Lu et al., 1994; Slater et al., 1995; Nievera et al., 2006), which recognizes and binds to newly replicated hemimethylated GATC sites that are clustered in *oriC* and in the *dnaA* promoter. High affinity recognition sites in *oriC* are not sequestered and can remain occupied throughout the cell cycle (Nievera et al., 2006), but low affinity *oriC* recognition sites and transcription of *dnaA* are blocked for about 1/3 of the cell cycle (Campbell and Kleckner, 1990). It is important to note that mechanisms of these types may play an important role in returning *oriC* to its correct bORC form, and thereby might turn out to be positive effectors of orisome assembly in addition to repressors.

In rapidly growing *E. coli*, where new rounds of DNA replication are triggered prior to the completion of previous rounds, there is insufficient new of DnaA–ATP synthesis to allow for proper initiation timing at all copies of *oriC*. A DnaA–ADP recharging system, dependent on two chromosomal loci termed DnaA reactivating sequences (DARSs) raises the levels of DnaA–ATP, although the exact mechanism remains unclear (Fujimitsu et al., 2009). DARS sites contain a specific arrangement of DnaA recognition sites, which must produce specific interactions between bound DnaA–ADP that promotes nucleotide exchange. Interestingly, DARS2 activity is regulated by both Fis and IHF (Kasho et al., 2014), the same DNA bending proteins that regulate orisome assembly. DARS are identified in a number of Gammaproteobacteria and this may be a common regulator for bacteria in this group that are capable of fast growth (Fujimitsu et al., 2009). There is also long standing evidence that DnaA interacts with the acidic phospholipids of the cytoplasmic membrane in *E. coli* (Crooke, 2001; Regev et al., 2012; Saxena et al., 2013) and like DARS, membranes recharge DnaA–ADP (Garner and Crooke, 1996).

In addition to the multiple mechanisms used to regulate DnaA–ATP levels and *oriC* accessibility, the existence of sites that preferentially bind DnaA–ATP suggest that the *oriC* sequence itself is an important component of the cell cycle timing mechanism for chromosome replication in *E. coli* and any other bacteria with similar sites. To test this idea, several obvious questions must be considered. Is the occupation of DnaA–ATP preferential recognition sites a rate-limiting step for initiation? If so, does each site play an equivalent role in determining the amount of DnaA–ATP needed for initiation, or is accessibility differentially regulated among sites? To answer these questions, it will be necessary to convert each discriminatory site into one that binds both nucleotide forms of DnaA equivalently. From preliminary studies, it appears that such conversion may alter the time of initiation in the cell cycle. For example, changing I2 and I3 into non-discriminatory sites on plasmid origins resulted in an origin that was more efficient that the chromosomal *oriC*, resulting in rapid integration of the cloned version into the host chromosome as a replacement for the wild-type *oriC* (Grimwade et al., 2007). Further analysis of mutations in individual and combinations of sites will determine their contribution to the cell cycle timing mechanism, but the possibility exists that all of the low affinity DnaA–ATP discriminatory sites in *E. coli oriC* can be changed into a form that binds DnaA–ADP, while maintaining orisome activity. If this is the case, it would provide strong evidence that that DnaA–ATP is not required for the assembly of the oligomeric filament along double-stranded DNA or even for initial DNA unwinding, but is required for correct cell cycle timing of initiation. In this scenario, the version of the DnaA–ADP oligomer assembled along *oriC* would be functionally equivalent to the DnaA–ATP version and it will be necessary to re-evaluate the pervading view of orisome assembly. It is important to note that regardless of the oligomers assembled along the arrayed low affinity sites, DnaA–ATP is also likely to be required later in orisome assembly for single-stranded DNA binding and helicase loading. Further studies should reveal the different roles of DnaA–ATP as a structural component and as a timing feature for the orisome.

## Orisomes are an Underexploited Drug Target

As a component of an essential machine for bacterial growth, and with regulatory proteins that are distinctly different from their eukaryotic counterparts, the orisome might be expected to be an excellent target for antibiotics. However, the orisome, like the replisome, remains under-exploited as a target for new drug development (Robinson et al., 2010). There are no known naturally occurring antibiotics that affect orisomes and perhaps this should be expected for an assemblage comprising a protein that is as highly conserved as DnaA. However, despite the dearth of orisome-specific inhibitors, DnaA activity has been be used as the basis for an antibiotic screen. Robust and high throughput *in vivo* assays (Fossum et al., 2008) were performed using *E. coli* conditional lethal, cold-sensitive strains (COS mutants) of DnaA which over-initiate new replication forks (Katayama and Kornberg, 1994). An inhibitor of orisome activity or replication fork movement will restore growth at the non-permissive temperature (Fossum et al., 2008). The strain also contained an alternative mode of chromosome replication to allow cell survival in the presence of an inhibitor that completely blocked DnaA activity. Using this assay, a novel benzazepine-derived, DNA gyrase inhibitor was identified from the Library of Pharmacologically Active Compounds (LOPACs; Johnsen et al., 2010). Although no direct inhibitors of DnaA were identified, over-expression of portions of the *E. coli* DnaA molecule (domain I or domain IV) were also shown to permit growth at low temperature in a similar assay, presumably by forming inactive hetero-oligomers and blocking orisome assembly (Weigel et al., 1999). Drugs modeled on these domains of DnaA may worth investigating in the future.

Successful orisome inhibitors may be most effective if they are directed toward specific stages or subassemblies of the orisome. Candidate sub-assemblies include the cross-strand DnaA–DnaA structures, promoted by DNA bending proteins like IHF, that may be “Achilles heel” stages that may require more time to assemble, or are less stable than other orisome assembly stages. These stages are likely already to be targets of cellular orisome assembly regulators such as DiaA (Ishida et al., 2004) or HobA (Zawilak-Pawlik et al., 2007).

**FIGURE 4:**
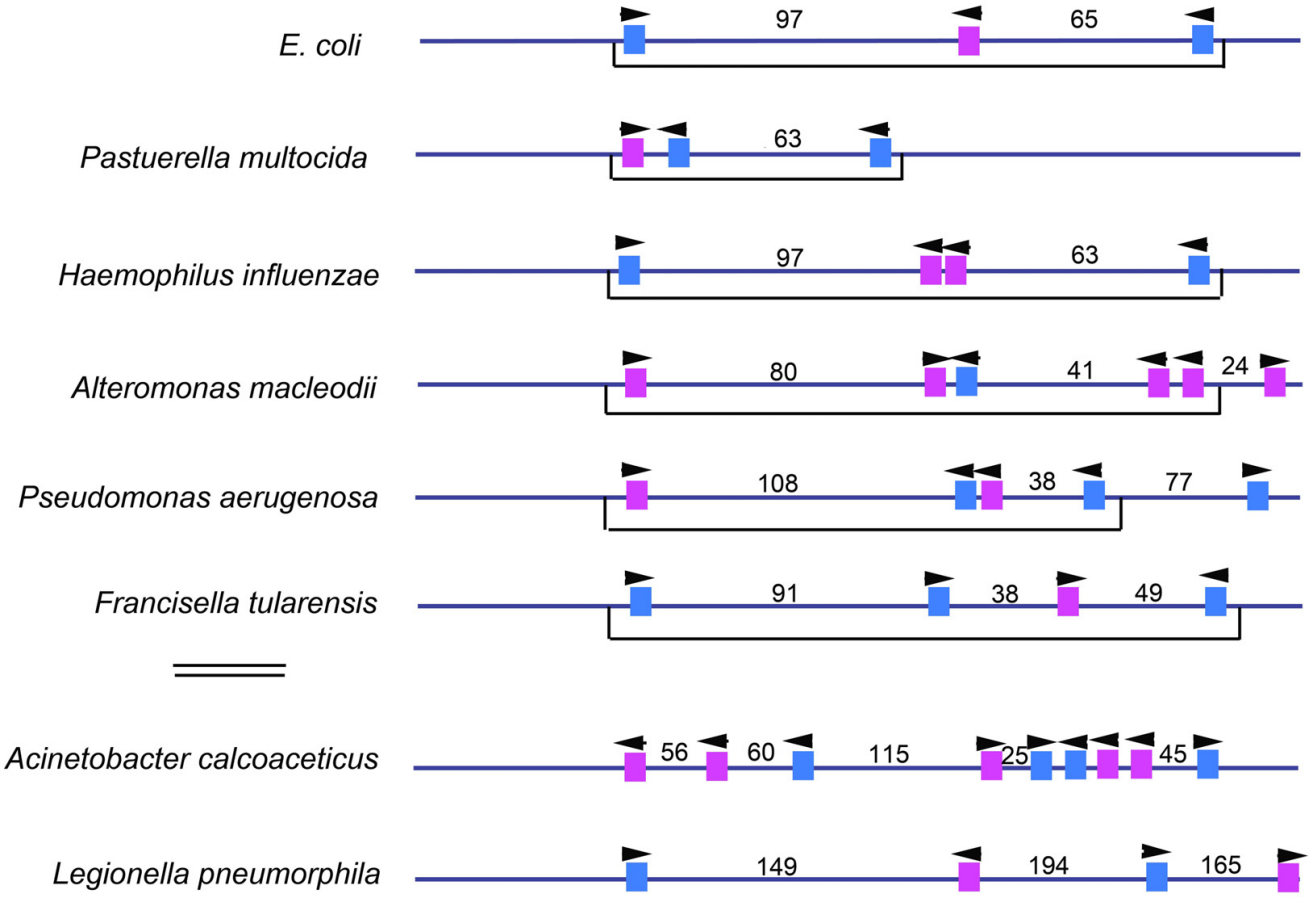
**Comparison of the number and placement of high affinity DnaA binding sites in *oriC*s in related members of the Gammaproteobacteria family.** The high affinity sites in the *oriC* regions of *E. coli* and several related bacterial types are shown. Blue rectangles indicate sites that match the consensus 5′-TTATCCACA, and the pink rectangles mark sites which deviate from this sequence at one or two bases. The arrowheads mark the presumptive orientation of the sites, and the numbers designate the number of base pairs in the gap regions between sites. The brackets below the maps show an *E. coli*-like arrangement of high affinity sites (see text for details). The two bacterial types below the double line are larger than those above, but it should be noted that the maps are not drawn to scale.

Another important aspect of targeting inhibitors of orisome assembly is to identify shared steps in orisome assembly among bacterial types. Fortunately, a large *oriC* database, DoriC, is available for comparative orisome analysis (Gao et al., 2013), but it is obvious from the diverse arrangements of consensus DnaA recognition sites that there are many different ways to assemble orisomes. Furthermore, there is a lack of information on low affinity DnaA recognition sites in bacteria other than *E. coli*. Although it appears that these sites exist in the well-studied origins of *Helicobacter*, *Mycobacteria*, *Bacillus,* and *Caulobacter* (Charbon and Lobner-Olesen, 2011; Taylor et al., 2011; Leonard and Mechali, 2013), for comparative analysis, it may be more informative to examine the origins of close relatives (for an example see, Shaheen et al., 2009). The *oriC* geography for relatives of *E. coli* (members of the Gammaproteobacteria) is shown in **Figure 4**. Even among the closely related members of this group, the arrangement, number, and orientation of consensus (high affinity) sites is variable. However, a shared, *E. coli*-like, motif appears with high affinity sites at the boundaries of gap regions (40–100 bp) where low affinity recognition sites would be expected to reside. This motif includes *E. coli*’s orientation of high affinity sites and may reflect the common assembly pattern of DnaA oligomers. Interestingly, some members (such as *Haemophilus influenza, Pseudomonas aerugenosa, and Alteromonas macleodii*) of this group carry extra high affinity sites that are centralized and extremely closely spaced. This arrangement suggests that in these origins, each DnaA oligomer may be anchored at its own high affinity site, rather than sharing one central site (R2) as is the case for *E. coli*. Other members (such as *Acinetobacter calcoaceticus*) have multiple high affinity sites downstream or upstream from the shared motif. Although the minimal requirement for high affinity sites remains to be determined for any member of this group other than *E. coli* (Kaur et al., 2014), additional high affinity sites may be required to build bORCs capable of forming larger or smaller DNA loops or to provide for synthesis of additional DnaA oligomers during staged orisome assembly. Some *oriCs* in this group (such as *A. calcoaceticus*) may also reflect a bipartite arrangement. Targeting these interesting versions of *oriC* that deviate from *E. coli* should provide valuable information to set the rules for orisome assembly and identify shared steps for inhibitor targeting.

It is worth noting that the *E. coli* recombineering strains mentioned above should be useful in developing heterologous systems to map DnaA-*oriC* interactions *in vivo* without having to study pathogenic organisms. Any replication origin can be introduced into the *E. coli* chromosome along with appropriate *dnaA* the gene as replacements for the *E. coli* versions. The heterologous origin does not need to be functional in these strains, since valuable information on orisome assembly can be obtained by examining origin binding to its cognate DnaA and, in the case of non-functional origins, where this process is halted. With a few modifications, a heterologous system of this type should also be useful for orisome inhibitor screening.

In summary, although more challenging experiments await, one can’t help but admire the versatility of the instruction set that is encoded into the bacterial origin of replication. Although some of its features are hidden within cryptic nucleotide sequence motifs, these instructions are sufficient to direct the addition of each DnaA subunit to produce staged orisome assembly, as well as encode recognition sites for proteins the produce regulatory switches for orisome assembly as well as binding sites for the regulatory proteins needed for origin resetting. Specific nucleotide sequences are likely to be involved in timing of orisome assembly in the cell cycle and it will be interesting to identify new roles for *oriC* sequences as different bacterial types are examined and compared. Clearly, despite the intense scrutiny that orisomes have received over the years, they still hold secrets that are worth uncovering.

## Conflict of Interest Statement

The authors declare that the research was conducted in the absence of any commercial or financial relationships that could be construed as a potential conflict of interest.
